# Anti-Tumor Effects of Engineered VNP20009-Abvec-Igκ-mPD-1 Strain in Melanoma Mice via Combining the Oncolytic Therapy and Immunotherapy

**DOI:** 10.3390/pharmaceutics14122789

**Published:** 2022-12-13

**Authors:** De-Xi Zhou, Xiao-He Wang, Xuan Xu, Wen-Jie Chen, Jing Wei, Ting-Tao Chen, Hong Wei

**Affiliations:** 1 School of Life Sciences, Nanchang University, Nanchang 330031, China; 2 Institute of Translational Medicine, Nanchang University, Nanchang 330031, China; 3 Precision Medicine Institute, The First Affiliated Hospital, Sun Yat-sen University, Guangzhou 510080, China

**Keywords:** VNP20009, mPD-1, bacterial therapy, melanoma

## Abstract

Programmed cell death protein 1/Programmed cell death ligand 1 (PD-1/PD-L1) immune checkpoint inhibitors are the most promising treatments for malignant tumors currently, but the low response rate limits their further clinical utilization. To address this problem, our group constructed an engineered strain of VNP20009-Abvec-Igκ-mPD-1 [V-A-mPD-1 (mPD-1, murine PD-1)] to combine oncolytic bacterial therapy with immunotherapy. Further, we evaluated its growth performance and mPD-1 expression ability in vitro while establishing the melanoma mice model to explore its potential anti-cancer effects in tumor therapy. Our results indicated that the V-A-mPD-1 strain has superior growth performance and can invade B16F10 melanoma cells and express PD-1. In addition, in the melanoma mice model, we observed a marked reduction in tumor volume and the formation of a larger necrotic area. V-A-mPD-1 administration resulted in a high expression of mPD-1 at the tumor site, inhibiting tumor cell proliferation via the down-regulation of the expression of rat sarcoma (Ras), phosphorylated mitogen-activated protein kinase (p-MEK)/MEK, and phosphorylated extracellular signal-regulated kinase (p-ERK)/ERK expression significantly inhibited tumor cell proliferation. Tumor cell apoptosis was promoted by down-regulating phosphoinositide 3 kinase (PI3K) and protein kinase B (AKT) signaling pathways, as evidenced by an increased Bcl-2-associated X protein/B cell lymphoma-2 (Bax/Bcl-2) expression ratio. Meanwhile, the expression levels of systemic inflammatory cytokines, such as interleukin-6 (IL-6), interleukin-1β (IL-1β), and tumor necrosis factor-α (TNF-α), were substantially reduced. In conclusion, our research demonstrated that V-A-mPD-1 has an excellent anti-tumor effect, prompting that the combined application of microbial therapy and immunotherapy is a feasible cancer treatment strategy.

## 1. Introduction

Melanoma is a highly aggressive malignant tumor that has its origin in melanocytes and often occurs on the skin, mucous membranes, and ocular choroids [1]. The number of new melanoma patients in 2020 is about 325,000 worldwide, accounting for 1.7% of total new cancer cases [2]. Studies indicated that lymphatic and hematological metastasis could occur in the early stages of melanoma formation [3], which has a survival rate of 5 years of only 10% [4], making it one of the malignancies that pose a critical threat to human health. Chemotherapy, radiotherapy, and surgery are conventional tumor treatments, but all have disadvantages such as toxicity to normal tissues or cells, the inefficiency of tumor targeting, and inability to penetrate tumor tissue, often leading to incomplete tumor killing [5]. Thus, there is an urgent demand to develop more efficient and specific strategies of treatment to be applied to overcome these limitations.

Bacterial therapy activates the anti-tumor ability of the immune system in the body by targeting tumor sites and has shown great potential in treating malignant tumors. As early as the late nineteenth century, William Coley injected inactivated *Streptococcal* and *Serratia marcescens* into tumor patients and observed the regression of tumors [6]. As research progressed, it has been observed that *Bifidobacterium bifidum* [7], *Salmonella* [8], *Listeria monocytogenes* [9], and *Escherichia coli* [10] preferentially accumulate and replicate in the oxygen-deprived zone of solid tumors and exhibit the inhibition of tumor growth. Among them, *Salmonella* possesses some advantages superior to other bacteria, including high tumor-specificity, deep-tissue penetrability, native bacterial cytotoxicity, and more concise genetic modification, and it has been receiving significant attention [11]. As a transgenic *S. typhimurium* strain, VNP20009 possesses excellent security profiles, including genetically stable attenuated virulence (*purI* gene deletion), reduced infectious shock potential (*msbB* gene deletion) and antibiotic susceptibility [12]. It has been effectively applied in phase I clinical trials in the medication of invasive melanoma and kidney cancer [13]. Several studies demonstrated that VNP20009 had been conceived and engineered as a tumor-targeting or drug-delivering vehicle displaying excellent therapeutic efficacies in mice models [14,15,16,17,18]. Furthermore, our previous work has demonstrated that modified VNP20009 expressing AIF protein exhibits attractive anti-tumor effects via a non-caspase-dependent pathway [19].

Immunotherapy has shown great potency in cancer treatment in recent years, in which immune checkpoint-based therapies have achieved unprecedented success in fighting cancer by revitalizing and enhancing T-cell responses [20]. PD-1/PD-L1 is an essential pair of immune checkpoints [21]. PD-1 on activated T cells can interact with PD-L1 overexpressed on cancer cells to induce the suppression of T-cell responses and the dysfunction of cytotoxic T cells [22]. Immune checkpoint inhibitors targeting the PD-1/PD-L1 interaction have been approved as basic clinical drugs in the treatment of melanoma, non-small cell lung cancer (NSCLC), renal carcinoma, and many other malignancies [23]. However, the response rates of PD-1/PD-L1 inhibitors in overall cases are unsatisfactory, restricting their extensive clinical practice [24]. Patients with practical treatment gain long-term survival [25], while others not only fail to respond to the treatment but also suffer from the immune side effects of the treatment [26]. Studies have shown that the integration of immunotherapy and other therapies could significantly improve anti-tumor efficacy and clinical response rates compared to monotherapy [27,28,29]. Particularly, Wang et al. generated an engineered oncolytic virus that co-expressed PD-L1 inhibitor and granulocyte-macrophage colony-stimulating factor (GM-CSF) and found that it activated tumor neoantigen-specific T-cell responses [30]. Based on previous studies, we raised the conjecture that the conjunction of oncolytic bacteria and immunotherapy could achieve equivalent anti-tumor effects.

In this study, we constructed an engineered VNP20009-Abvec-Igκ-mPD-1 strain that combines both the effects of VNP20009 (tumor colonization and killing ability) and mPD-1 single-chain antibody (alleviation of T cell suppression). Next, we subcutaneously injected B16F10 cells into mice to construct melanoma mice models and explored the potential molecular mechanism for the effect of the engineered bacteria on tumor cell proliferation and apoptosis by H&E staining, immunohistochemistry, and Western blotting. We aim to develop a novel therapeutic tool for clinical research with significant effects and fewer side effects while providing a theoretical basis and data support for the improvement of engineered biologics.

## 2. Materials and Methods

### 2.1. Construction of Engineered Strain and Evaluation In Vitro

To construct the recombinant plasmid AbVec-Igκ-mPD-1, we insert the PD-1 gene (*Mus musculus*), synthesized by Sangon Biotech (Shanghai, Co., Ltd., Shanghai, China), into AbVec-Igκ (FitGene Biotechnology (Guangzhou) Co., Ltd., Guangzhou, China) eukaryotic expression vector. The engineered VNP20009-AbVec-Igκ (V-A) and VNP20009-AbVec-Igκ-mPD-1 (V-A-mPD-1) strains were constructed by electroporation via transforming the empty plasmid and the recombinant plasmid into the attenuated *Salmonella typhimurium* VNP20009 (ATCC 202165), respectively. After that, it is tested for growth curves and evaluated for plasmid stability.

The growth curve was determined using the turbidimetric method. The V-A and V-A-mPD-1 strains were activated overnight, 1 mL was inoculated into 100 mL of LB liquid medium, incubated at 37 °C and 150 rpm shaking conditions, and the growth curves were measured at 0, 2, 4, 6, 8, 10, 12, 14, 16, 18, 20, 22, and 24 h, respectively. The growth curves of the suspension were then plotted using the OD_600_ value as the vertical coordinate and the incubation time as the horizontal coordinate [31].

The V-A-mPD-1 strain was inoculated overnight in LB liquid medium without antibiotics and then passaged for 25 generations in an LB medium with 50 µg/mL Ampicillin (Amp) and without Amp. The number of spot plates in the LB plates were counted every three generations; the culture in the medium without Amp does not require repeated counting. Plasmid stability = the number of viable bacteria in every third generation/the number of viable bacteria in the first generation [32].

### 2.2. The Ability of V-A-mPD-1 to Enter B16F10 and Express PD-1

The B16F10 mouse melanoma cell line was purchased from Fuxiang Biological Company (Shanghai, China) and cultured in RPMI1640 medium containing 10% fetal bovine serum (FBS) and 1% penicillin at 37 °C and 5% CO_2_ to the logarithmic growth phase. The cells were collected and resuspended in PBS for subsequent experiments.

We inoculated B16F10 cells at 1 × 10^5^ cells/well in 6-well plates and incubated for 24 h in 3 mL RPMI1640 medium containing 10% FBS and 1% penicillin/streptomycin. After the cells had grown to 70% density, the RPMI1640 medium was refreshed without antibiotics and divided into the following three groups: (A) control group (B16F10 cells only); (B) V-A+B16F10 cells group; (C) V-A-mPD-1+B16F10 cells group (1:100 ratio of bacteria to cells). The V-A and V-A-mPD-1 strains were then incubated with the cells at 37 °C and 5% CO_2_ for 2 h. After 2 h, the cells were washed three times with sterile PBS and then replaced with resistant RPMI1640 medium for a further 3, 6, and 10 h, respectively. Finally, after three washes at the different time points mentioned above, the cell–bacteria co-cultures were collected for the subsequent experiments. The number of viable bacteria was detected using a viable count method to observe whether bacteria had entered the cells [33]. Meanwhile, the proteins were extracted from the bacterial cell co-cultures for the Western blotting experiments to explore the expression of V-A-mPD-1 in B16F10.

### 2.3. Development and Treatment of Melanoma Mice Model

The research protocol and animal experiments for this study were approved by the Laboratory Animal Ethics Committee of Nanchang Royo Biotechnology Co., Ltd., Nanchang, China (Approval number: RyE2021070914).

Forty-two C57BL/6 wild-type male mice aged six to eight weeks, weighing 18–20 g, of the specific-pathogen free (SPF) level, were purchased from Hunan SJA Laboratory Animal Co., Ltd. All of the mice were housed under an SPF environment (humidity 50 ± 15%, temperature 22 ± 2 °C, 12/12 light-dark cycle) with ad libitum access to standard food and water. After the adaptation of the mice for 1 week, six were randomly selected as the normal group (C), and the remaining mice were then subcutaneously injected with 5 × 10^5^ B16F10 cells in the dorsal region. After cell inoculation for 10 days, the mice were randomly divided into three groups according to tumor size (tumor volume at around 100 mm^3^). (1) Model (Sterile saline injected intraperitoneally on day 13 after tumor inoculation, every three days, *n* = 12); (2) V-A (5 × 10^3^ CFUs V-A cells were injected intraperitoneally on day 13 after tumor inoculation [34], injection every three days, *n* = 12); (3) V-A-mPD-1 (5 × 10^3^ CFUs V-A-mPD-1 cells were injected intraperitoneally on day 13 after tumor inoculation, injection every three days, *n* = 12).

On day 25, the mice were anesthetized with 2% isoflurane and then euthanized by cervical dislocation. A portion of the tumor tissue (except the C group) was collected and fixed in 4% paraformaldehyde, and the remainder was frozen at −80 °C. Tumor volume = length × width^2^ × 0.5.

### 2.4. Pathological Histology

The fresh melanoma tissue was paraffin-embedded and cut into 5 µm sections. After treatment with xylene and decreasing grades of ethanol, an examination of tumor slides stained with hematoxylin and eosin (H&E) under a microscope was performed.

Immunohistochemical analysis was performed according to standard publication protocols [35]. The respective antibodies used are an anti-vascular endothelial growth factor (VEGF) antibody (CST, 9698s) and an anti-proliferating cell nuclear antigen (PCNA) antibody (CST, 13110s).

### 2.5. Real-Time Fluorescence Quantitative PCR

For the extraction of RNA, serum was added into the TRIzol reagent (Invitrogen, Waltham, MA, USA) at a ratio of 1:3 and hemolyzed at room temperature for 10 min, after which the concentration and purity of the RNA were tested using a NanoDop spectrophotometer. The RNA was reverse transcribed using the Takara PrimeScript RT reagent kit to remove genomic DNA and obtain cDNA. Quantitative PCR amplifications were performed using the 7900HT rapid real-time PCR system (ABI) and SYBR Green fluorescent dye. The procedure for amplification was configured to start at 95 °C for 10 min, followed by deterioration at 95 °C for 30 s, then annealing at 60 °C for 30 s and extending at 72 °C for 30 s, for 40 cycles. The relative levels of the target genes were analyzed using the 2^−ΔΔCt^ method with GAPDH as the internal reference gene. The primer sequences used in this experiment are listed below: IL-6: 5′-GGAAATCGTGGAAATGAG-3′ (forward) and 5′-GCTTAGGCATAACGCACT-3′ (reverse); IL-1β: 5′-GTGTCTTTCCCGTGGACCTTC-3′ (forward) and 5′-TCATCTCGGAGCCTGTAGTGC-3′ (reverse); TNF-α: 5′-GTGGAACTGGCAGAAGAGGCA-3′ (forward) and 5′-AGAGGGAGGCCATTTGGGAAC-3′ (reverse) and GAPDH: 5′-CTCGTGGAGTCTACTGGTGT-3′ (forward) and 5′-GTCATCATACTTGGCAGGTT-3′ (reverse).

### 2.6. Western Blotting

The melanoma tissue was weighed to 100 mg, and 1 mL of radioimmunoprecipitation assay (RIPA) lysis buffer (Solarbio, R0010) containing 10 µL of protease inhibitor and phosphatase inhibitor, respectively, was added to it and placed on ice for sonication to lyse it well. The homogenate was centrifuged at 8000× *g* for 15 min at 4 °C, and the supernatant was collected for protein concentration determination by BCA (Thermo Fisher, A53226). The protein samples were thoroughly mixed with protein-loading buffer and thermally denatured by boiling at 100 °C for 10 min. Each group was then separated by gel electrophoresis (SDS-PAGE) at a protein-loading volume of 60 µg/lane (the concentration of the separation gel was determined by the molecular weight of the target protein) and transferred to the appropriate size nitrocellulose membrane (Millipore, Darmstadt, Germany). The PVDF membranes were then closed at room temperature for 1 h using TBST containing 0.1% Tween-20 and 5% skim milk. A diluted primary antibody was added to the membranes and incubated overnight at 4 °C. Information on the primary antibodies used in the study is as follows: anti-β-actin (Abcam, ab8226), anti-PD-1 (Proteintech, 18106-1-AP), anti-AΚT (CST, 9272s), anti-p-AΚT (CST, 4060s), anti-PI3K (Abcam, ab151549), anti-p-PI3K (CST, 17366s), anti-Bax (CST, 2772s), anti-Bcl-2 (Proteintech, 12789-1-AP), anti-Ras (CST, 3965s), anti-MEK (CST, 4694s), anti-p-MEK (CST, 9154s), anti-ERK (CST, 4695s), and anti-p-ERK (CST, 8544s). After three washes with TBST, the membranes were incubated for 1 h using horseradish peroxidase (HRP)-conjugated goat anti-rabbit or goat anti-mouse secondary antibodies, followed by the development of the membranes by enhanced chemiluminescence. The intensity of the immunoreactive bands was quantified by measuring them with ImageJ software.

### 2.7. Statistical Analysis

All of the data for statistical analysis were analyzed using Prism software version 6.01 (GraphPad Software, San Diego, CA, USA). The two-tailed Student’s *t*-test was applied to analyze the differences between the two groups. One-way ANOVA or two-way ANOVA was used to assess the differences between three or more groups, followed by Tukey’s multiple comparison post hoc test. All of the data for the experimental results are presented as mean ± standard deviation (SD). * *p* < 0.05 was considered statistically significant.

## 3. Results

### 3.1. Evaluation of V-A-mPD-1 In Vitro

**Figure 1:**
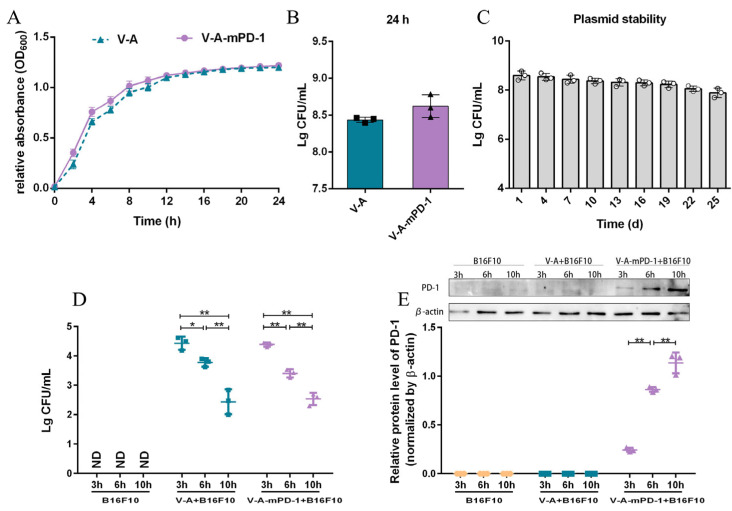
Evaluation of V-A-mPD-1 in vitro. (**A**) Growth curves of V-A and V-A-mPD-1. (**B**) The number of viable bacteria corresponding to 24 h incubation with V-A and V-A-mPD-1. (**C**) Plasmid stability test of V-A-mPD-1. (**D**) The quantity of the living bacteria in the B16F10 group, V-A+B16F10 group and V-A-mPD-1+B16F10 group at 3 h, 6 h and 10 h. (**E**) The expression levels of the mPD-1 protein in the B16F10 group, V-A+B16F10 group and V-A-mPD-1+B16F10 group were detected by Western blotting at 3 h, 6 h, and 10 h. V-A, VNP20009-Abvec-Igκ; V-A-mPD-1, VNP20009-Abvec-Igκ-mPD-1. Two-tailed t-test for A and B, one-way ANOVA (**C**) or two-way ANOVA (**D**,**E**) and Tukey’s multiple comparison. Values are presented as means ± SD (*n* = 3). * *p* < 0.05; ** *p* < 0.01.

Firstly, we monitored the growth of the empty plasmid-bearing VNP20009 strain (V-A) and the mPD-1-expressing VNP20009 strain (V-A-mPD-1) over a 24 h period using turbidimetry. As shown in Figure 1A, the V-A and V-A-mPD-1 strains entered the logarithmic growth period at 2 h and the stable growth period after 16 h. We found no difference in the growth characteristics between V-A and V-A-mPD-1. At 24 h, the viable count of strain V-A was about 2.8 × 10^8^ CFU/mL, and that of V-A-mPD-1 was about 4.4 × 10^8^ CFU/mL (Figure 1B). After that, we evaluated the plasmid stability of V-A-mPD-1, which reached 91.8% after passing the plasmid once a day for up to 25 generations (Figure 1C). Next, we examined the expression of mPD-1 in a eukaryotic system, as shown in Figure 1D,E, the V-A or V-A-mPD-1 strain actively invaded B16F10 cells, and bacteria viability (CFU) was significantly decreased in a time-dependent manner, accompanied by up-regulation of mPD-1 expression in the V-A-mPD-1 strain. All these results indicated that the engineered bacterium V-A-mPD-1 had good growth characteristics and invasive properties.

### 3.2. V-A-mPD-1 Administration Inhibits Melanoma Growth in Mice

**Figure 2:**
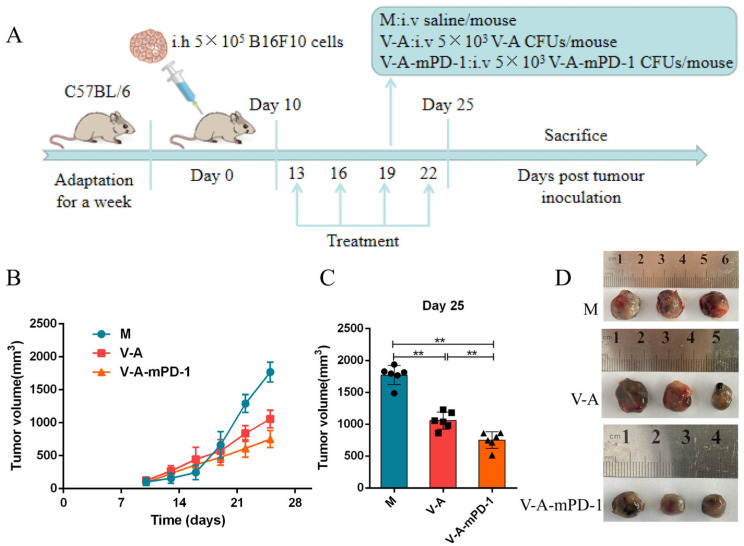
Inhibition of tumor growth in mice melanoma model by the anti-tumor effect of the V-A-mPD-1 strain. (**A**) Treatment schedule for mice bearing B16F10 melanoma models. (**B**) Changes in tumor volume over time in M, V-A, V-A-mPD-1 group. (**C**) Tumor size of mice in each group at day 25. (**D**) Images of tumor in M, V-A, V-A-mPD-1 group at day 25. M group, the melanoma mice were treated with sterilized saline; V-A group, the melanoma mice were intraperitoneally injected with VNP20009-Abvec-Igκ strain; V-A-mPD-1 group, the melanoma mice were given VNP20009-Abvec-Igκ-mPD-1 strain. Two-way ANOVA and Tukey’s multiple comparison for B and C. Data are presented as means ± SD (*n* = 6). ** *p* < 0.01.

PD-1 released from V-A-mPD-1 can compete with T cells to bind PD-L1 on the surface of tumor cells, thereby reducing tumor immune escape as well as facilitating cancericidal immune action. To investigate the in vivo anti-tumor effects of V-A-mPD-1, we established tumor-bearing mice models of melanoma to assess the anti-tumor effect of the engineered bacterium V-A-mPD-1 (Figure 2A). As shown in Figure 2B, in comparison to the model group, the tumor volume in the V-A and V-A-mPD-1 groups was significantly reduced, and the effect of V-A-mPD-1, in particular, was more pronounced. Specifically, the V-A-mPD-1 group showed good anti-tumor effects from day 19 onwards, and tumor growth was markedly delayed (V-A vs. M, 571.5 mm^3^ vs. 663.8 mm^3^, V-A-mPD-1 vs. M, 475.7 mm^3^ vs. 663.8 mm^3^, *p* < 0.05). On the 25th day, V-A-mPD-1 showed potent anti-tumor effects compared to the M and V-A groups (V-A-mPD-1 vs. M, 750.8 mm^3^ vs. 1770 mm^3^, *p* < 0.01, V-A-mPD-1 vs. V-A, 750.8 mm^3^ vs. 1057 mm^3^, *p* < 0.01) (Figure 2C,D).

### 3.3. V-A-mPD-1 Inhibits Melanoma Growth by Suppressing Tumor Proliferation

**Figure 3:**
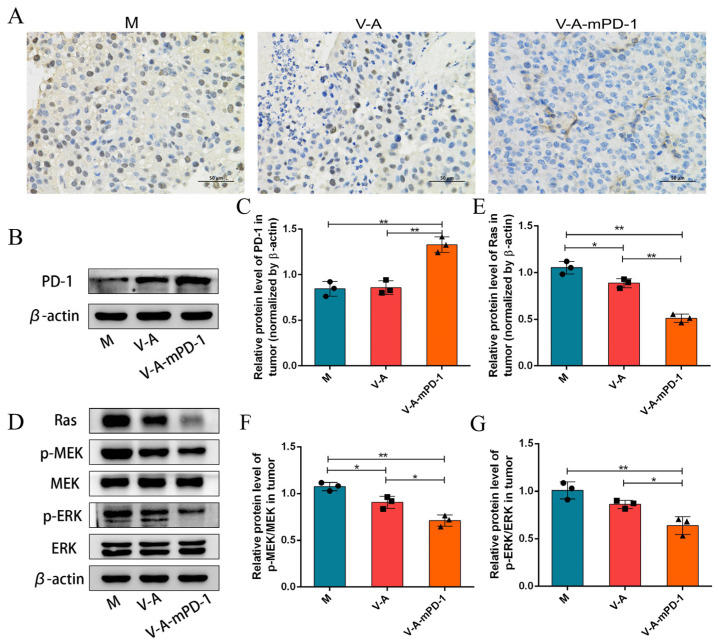
Proliferation of murine melanoma tumors is clearly inhibited by colonization of tumors with the V-A-mPD-1 strain. (**A**) Immunohistochemical detection of PCNA level in tumor tissue (400×). (**B**) Western blotting analysis of PD-1 expression in tumor tissues. (**C**) Relative expression of PD-1 was quantified by ImageJ, and β-actin was used as an internal control. (**D**) Western blotting analysis of Ras, p-MEK, MEK, p-ERK, ERK expression in tumor tissues. The relative expressions of (**E**) Ras, (**F**) p-MEK/MEK, (**G**) p-ERK/ERK were quantified by ImageJ. β-actin was used as an internal control. One-way ANOVA and Tukey’s multiple comparison for C, E, F, and G. Data are presented as means ± SD (*n* = 3). * *p* < 0.05, ** *p* < 0.01.

For the evaluation of the anti-tumor effect of V-A-mPD-1, we performed immunohistochemical assays, and the outcomes revealed that the V-A-mPD-1 strain markedly inhibited tumor cell proliferation by suppressing the expression of proliferating cell nuclear antigen (PCNA) (Figure 3A). Then, we examined the levels of PD-1 expressed in the tumors of each group of mice. As shown in Figure 3B,C, the PD-1 protein levels were substantially up-regulated in the V-A-mPD-1 group compared to the M and V-A groups (V-A-mPD-1 vs. M, 1.33 vs. 0.8456, *p* < 0.01, V-A-mPD-1 vs. V-A, 1.33 vs. 0.8579, *p* < 0.01). In addition, we determined the related proteins in the tumor proliferation signaling pathway Ras/Raf/MEK/ERK by Western blotting analysis, which is particularly activated in melanoma by BRAF mutation B-Raf^V600E/K^. The results of the study showed that compared to the M group, the expression of Ras (0.8865, 0.5095, respectively), p-MEK/MEK (0.9066, 0.7104, respectively), and p-ERK/ERK (0.8612, 0.6403, respectively) were significantly down-regulated in the V-A and V-A-mPD-1 groups (except for p-ERK/ERK in the V-A group), and the down-regulation trend was more obvious in the V-A-mPD-1 group in particular (Figure 3D–G). All these results suggest that the inhibition of tumor cell proliferation may be mediated by the presence of PD-1.

### 3.4. V-A-mPD-1 Promotes Tumor Necrosis and Reduces Systemic Inflammatory Responses in Mice

**Figure 4:**
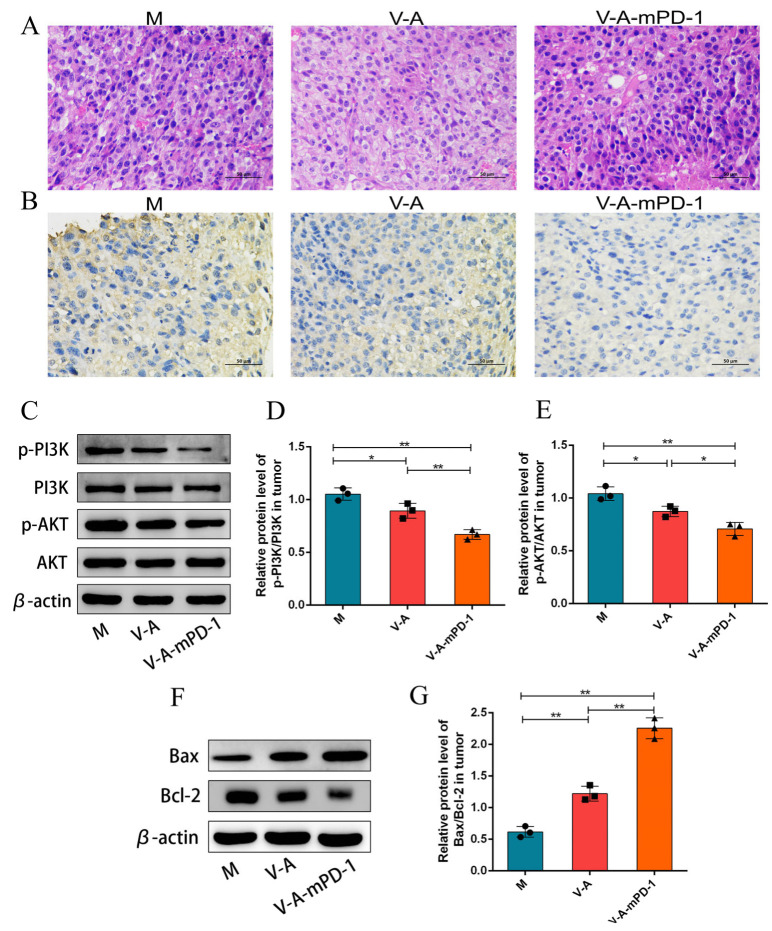
V-A-mPD-1 exerts tumor-killing effects by inducing apoptosis. (**A**) H&E staining images of tumor tissues (400×); (**B**) Immunohistochemistry of VEGF expression was determined in melanoma tumors (400×); (**C**) p-PI3K, PI3K, p-AKT and AKT expressions were analyzed using Western blotting on the tumors; Relative expressions of (**D**) p-PI3K, PI3K and (**E**) p-AKT, AKT in (**C**); (**F**,**G**) Relative expression of Bax/Bcl-2 was analyzed by Western blotting in tumors. M, the melanoma model group; V-A, the V-A treatment group; V-A-mPD-1, the V-A-mPD-1 treatment group. One-way ANOVA and Tukey’s multiple comparison for D, E, and G. Data are presented as means ± SD (*n* = 3). * *p* < 0.05, ** *p* < 0.01.

**Figure 5:**
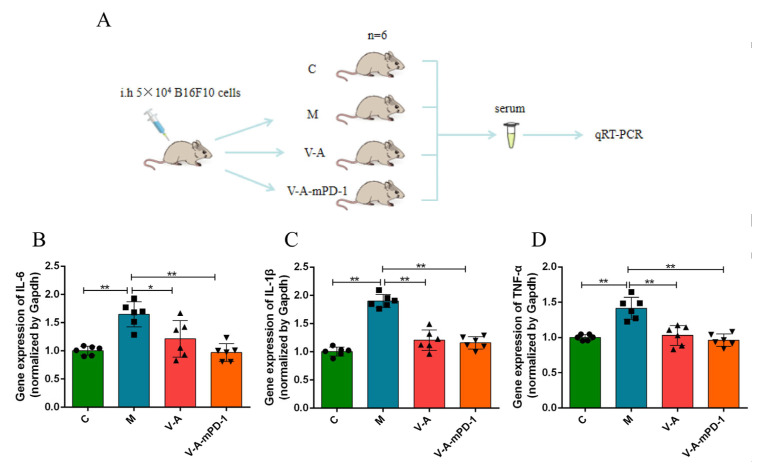
V-A-mPD-1 has the potential to reduce systemic inflammation in melanoma mice. (**A**) The experimental scheme of q-PCR. (**B**–**D**) Pro-inflammatory cytokines IL-6, IL-1β, and TNF-α were detected in serum at the gene level by q-PCR. C, The normal control group; M, the melanoma model group; V-A, the V-A treatment group; V-A-mPD-1, the V-A-mPD-1 treatment group. One-way ANOVA and Tukey’s multiple comparison for B–D. Data are presented as means ± SD (*n* = 6). * *p* < 0.05, ** *p* < 0.01.

To examine the effect of V-A-mPD-1 administration on apoptosis, we performed H&E staining, and the findings displayed dense and homogeneous tumor cells with deeply stained nuclei in the M group, while some necrotic tumor cells were detected in the V-A group and large areas of nuclear fission, fragmentation, and lysis necrosis were detected in the V-A-mPD-1 group (Figure 4A). Using immunohistochemical analysis, we found that the V-mPD-1 strain inhibited the expression of vascular endothelial growth factor (VEGF), which represented a trend towards increased neointima formation and microvessel density, suggesting that angiogenesis at the tumor site was inhibited (Figure 4B). The apoptotic pathway mediated by Bcl-2 family proteins was then further investigated. As shown in Figure 4C–E, V-A-mPD-1 greatly reduced the expression of p-PI3K/PI3K (V-A-mPD-1 vs. M, 0.6698 vs. 1.053, *p* < 0.01, V-A-mPD-1 vs. V-A, 0.6698 vs. 0.8947, *p* < 0.01) and p-AKT/AKT (V-A-mPD-1 vs. M, 0.7083 vs. 1.041, *p* < 0.01, V-A-mPD-1 vs. V-A, 0.7083 vs. 0.8739, *p* < 0.05) compared with the M group and the V-A group. Meanwhile, compared with the M group, the Bax/Bcl-2 expression ratio of the V-A group and the V-A-mPD-1 group was significantly increased (1.221 and 2.255, respectively) (Figure 4F,G). The above results suggest that V-A-mPD-1 could activate PD-1-mediated tumor cell apoptosis strongly. Finally, we measured the expression levels of pro-inflammatory factors in the serum of each group of mice by q-PCR to investigate the effect of the administration of the engineered bacteria on the level of systemic inflammation in mice and compared the expression levels with those of the normal group of mice. The results showed that V-A and V-A-mPD-1 significantly reduced the relative expression of pro-inflammatory factors IL-1β (1.206, 1.159, respectively), IL-6 (1.212, 0.9695, respectively), and TNF-α (1.029, 0.9621, respectively) in the serum compared to the M group, with the levels in the V-A-mPD-1 group being closer to those in the C group in particular (Figure 5).

## 4. Discussion

Cancer is the second most lethal disease in the world after ischemic heart disease. According to the World Health Organization (WHO), it is predicted to be the primary cause of death by 2060, with around 18.63 million deaths [36]. Conventional cancer treatments have limited efficacy, and finding a therapy that is highly selective for tumors and has low toxicity to normal tissue is one of the challenges faced by oncology researchers. Studies have demonstrated that the rapid growth of tumor cells and the poor vascularization and heterogeneity of tumors result in a hypoxic, eutrophic, and immunosuppressed state of the microenvironment within solid tumors [37]. The ability of anaerobic bacteria to target and colonize the hypoxic regions of solid tumors provides an important therapeutic opportunity for cancer treatment [38]. VNP20009 is an attenuated strain of the genus *Salmonella* that, as a parthenogenic anaerobic bacterium, grows in both the viable and necrotic zones of tumors and displays anti-cancer effects [39]. However, studies have shown that bacteria with inherent anti-tumor properties cannot wholly eradicate tumors on their own and must be combined with other therapies to increase the therapeutic effect.

To date, PD-1/PD-L1 immune checkpoint inhibitors, a branch of immunotherapy, have made significant progress in cancer treatment [40]. However, the inhibitors suffer from adverse effects such as low response rates and significant side effects due to individual differences. Thus, researchers are exploring the possibility of combining anti-PD-1/PD-L1 therapy with other treatments in an attempt to produce synergistic effects. A phase Ib clinical trial indicated that the combination of talimogene laherparepvec’s lysoviral with anti-PD-1 therapy produced high response rates in patients with advanced melanoma [41]. Therefore, combining bacterial therapy with checkpoint inhibitor therapy in this study is reasonable to maximize the anti-tumor effects.

In our research, we constructed an engineered strain of VNP20009-Abvec-Igk-mPD-1 (V-A-mPD-1) and evaluated the growth performance of the engineered strain and its ability to express mPD-1 in vitro through animal experiments. Growth curve experiments and plasmid stability experiments demonstrated that the engineered bacteria had excellent growth ability and stability. Further, we co-cultured the V-A-mPD-1 strain with B16F10 cells and observed that the bacteria could invade B16F10 cells and stably express the mPD-1 protein. The above experimental results indicated that V-A-mPD-1 was successfully constructed and had potential invasiveness (Figure 1).

Subsequently, we established the melanoma mice model to assess whether V-A-mPD-1 has anti-tumor efficacy in vivo. As shown in Figure 2, both V-A and V-A-mPD-1 significantly suppressed the growth of tumors compared to the model group, with the effect of V-A-mPD-1 being more pronounced. This suggests that VNP20009 itself has tumor-killing properties and that its ability to express mPD-1 simultaneously may enhance this effect. We will now clarify the possible mechanism of this phenomenon.

The proliferation of tumor cells can be used as a prognostic indicator to assess the extent of tumor invasion for the purpose of monitoring and predicting the effectiveness of anti-tumor therapy [42]. Our immunohistochemical results showed significant downregulation in the positive rate of PCNA in the V-A-mPD-1 group. PCNA can be used as a proliferation marker in cancer diagnosis to assess the proliferative activity of tumor cells [43], thus indicating that the administration of engineered bacteria can inhibit tumor proliferation. Furthermore, V-A-mPD-1 significantly reduced the protein expression of Ras, p-MEK/MEK, and p-ERK/ERK. It was shown that the proliferation of melanoma is primarily regulated by the RAS/RAF/MEK/ERK signaling pathway. Upon activation of this pathway by oncogenic mutations (e.g., BRAF), signals are transmitted from cell surface receptors to transcription factors via a series of protein phosphorylation, thereby regulating apoptosis and cell-cycle progression [44,45]. This further confirms that V-A-mPD-1 may inhibit tumor growth by suppressing cell proliferation (Figure 3).

Studies have revealed that the abnormal proliferation of tumors is often accompanied by features such as low cell differentiation [46], chromosomal abnormalities [47], and the nuclear heterogeneity of cells [48]. Our H&E staining results showed significant tumor necrosis, nuclear solidification, and fragmentation in the V-A-mPD-1 group when contrasted with the control group. In addition, VEGF was significantly down-regulated in the V-A-mPD-1 group. The VEGF is an important pro-angiogenic factor that is essential for the angiogenesis and proliferation of vascular endothelium [49]. This suggests that V-A-mPD-1 effectively inhibited angiogenesis within the tumor. Then, to further investigate whether V-A-mPD-1 treatment induced apoptosis in tumor cells, we detected the expression of proteins related to the growth and apoptosis signaling pathways. V-A-mPD-1 significantly down-regulated p-PI3K/PI3K and p-AKT/AKT expression, and the Bax/Bcl-2 ratio was up-regulated. It is well known that the PI3K/AKT signaling pathway has been shown to be extensively involved in the regulation of a variety of biological processes and is a major pathway for promoting cell growth and proliferation [50]. Among these, AKT induces the expression of genes encoding anti-apoptotic proteins and is an essential inhibitor of apoptosis in vivo [51]. Bcl-2, a signaling protein downstream of the PI3K/AKT pathway, acts as an antagonist with the Bax to regulate the development of apoptosis [52]. Studies have shown that PI3K/AKT phosphorylation inhibits Bax activity and promotes the release of Bcl-2 [53,54], which confirms the potential therapeutic effect of this bacterium as an anti-cancer drug (Figure 4).

Finally, we assessed the level of systemic inflammation in mice. V-A-mPD-1 administration was accompanied by a reduction in the inflammatory cytokines IL-6, IL-1β, and TNF-α. It was shown that immunosuppressive molecules secreted by tumor cells could promote the secretion of IL-10, IL-6, TNF-α, and COX-2 inflammatory mediators by macrophages, mast cells, tumor-associated fibroblasts, endothelial cells, neutrophils, NK cells, and DCs that are enrolled in the tumor tissue, thus creating a vicious circle that results in tumor cells escaping from the immune system [55]. Furthermore, an in vitro study also demonstrated that the addition of TNF-α, IL-1β, and IL-6 factors to the conditioned medium of activated macrophages significantly triggered the proliferation and immigration of human colon carcinoma cells [56]. Thus, the reduced levels of inflammatory factors in the V-A-mPD-1 group further confirmed its anti-tumor effects (Figure 5).

## 5. Conclusions

In short, our studies reveal that V-A-mPD-1 administration mediates the inhibition of tumor cell proliferation and the onset of apoptosis through the virulence of the bacteria themselves and the release of mPD-1. It is accompanied by a decrease in the level of serum inflammatory factors, providing a new idea for an anti-tumor strategy combining bacteriotherapy and immunotherapy. However, we have only explored the short-term effects of V-A-mPD-1 on the growth of this tumor by establishing the mice melanoma model. In the future, further studies are needed regarding whether the excessive release of mPD-1 affects the body’s immune environment and the specific mechanisms underlying the anti-tumor effects of engineered V-A-mPD-1.

## Data Availability

All the data from the study are available from the corresponding author upon reasonable request.
